# Prognostic and treatment predictive significance of SATB1 and SATB2 expression in pancreatic and periampullary adenocarcinoma

**DOI:** 10.1186/s12967-014-0289-8

**Published:** 2014-10-17

**Authors:** Jacob Elebro, Margareta Heby, Alexander Gaber, Björn Nodin, Liv Jonsson, Richard Fristedt, Mathias Uhlén, Karin Jirström, Jakob Eberhard

**Affiliations:** Department of Clinical Sciences Lund, Oncology and Pathology, Lund University, Skåne University Hospital, 221 85 Lund, Sweden; Science for Life Laboratory, Royal Institute of Technology, 171 21 Stockholm, Sweden; School of Biotechnology, AlbaNova University Center, Royal Institute of Technology, 106 91 Stockholm, Sweden

**Keywords:** Periampullary adenocarcinoma, Pancreatic cancer, Immunohistochemistry, Biomarkers, Prognosis, Treatment prediction

## Abstract

**Background:**

Pancreatic cancer and other pancreaticobiliary type periampullary adenocarcinomas have a dismal prognosis even after resection and neoadjuvant chemotherapy. Intestinal type periampullary adenocarcinomas generally have a better prognosis, but little is known on optimal neoadjuvant and adjuvant treatment. New prognostic and treatment predictive biomarkers are needed for improved treatment stratification of patients with both types of periampullary adenocarcinoma. Expression of the Special AT-rich sequence-binding protein 1 (SATB1) has been demonstrated to confer a worse prognosis in several tumour types, whereas its close homologue SATB2 is a proposed diagnostic and favourable prognostic marker for colorectal cancer. The prognostic value of SATB1 and SATB2 expression in periampullary adenocarcinoma has not yet been described.

**Methods:**

Immunohistochemical expression of SATB1 and SATB2 was analysed in tissue microarrays with primary tumours and a subset of paired lymph node metastases from 175 patients operated with pancreaticoduodenectomy for periampullary adenocarcinoma. Kaplan-Meier and Cox regression analysis were applied to explore the impact of SATB1 and SATB2 expression on recurrence free survival (RFS) and overall survival (OS).

**Results:**

Positive expression of SATB1 was denoted in 16/106 primary pancreatobiliary type tumours and 11/65 metastases, and in 15/63 primary intestinal type tumours and 4/26 metastases, respectively. Expression of SATB1 was an independent predictor of a significantly shorter RFS and OS in pancreatobiliary type, but not in intestinal type adenocarcinomas. Moreover, SATB1 expression predicted an improved response to adjuvant chemotherapy in both tumour types. SATB2-expression was seen in 3/107 pancreatobiliary type primary tumours, and in 8/61 intestinal type primary tumours. The small number of cases with positive SATB2 expression did not allow for any firm conclusions on its prognostic value.

**Conclusions:**

These findings demonstrate the potential utility of SATB1 as a prognostic and predictive biomarker for chemotherapy response in both intestinal type and pancreatobiliary type periampullary adenocarcinomas, including pancreatic cancer.

**Electronic supplementary material:**

The online version of this article (doi:10.1186/s12967-014-0289-8) contains supplementary material, which is available to authorized users.

## Background

Periampullary adenocarcinomas encompass tumours originating in or adjacent to the ampulla of Vater; pancreatic cancer, distal bile duct cancer, ampulla of Vater carcinoma and carcinoma of the periampullary duodenum. Pancreatic cancer is the most common type of periampullary adenocarcinoma, but only a minority can be resected with a curative intent, due to either locally advanced growth or distant metastases at presentation. There are two major morphological types of periampullary adenocarcinomas, which have different prognosis and receive different chemotherapy. Pancreatobiliary type (PB-type) adenocarcinomas include pancreatic cancer, distal bile duct cancer, and some of the ampullary carcinomas. They have a dismal prognosis even after resection and adjuvant gemcitabine-based chemotherapy. Intestinal type (I-type) periampullary adenocarcinomas include duodenal carcinoma and some of the ampullary carcinomas. They have a better prognosis but little is known on risk stratification and optimal chemotherapy [1,2]. Hence, new biomarkers are needed to better stratify both PB-type and I-type periampullary adenocarcinomas according to risk and expected response to treatment.

Special AT-rich sequence-binding protein 1 (SATB1) is a genome organizing protein which regulates region-specific epigenetic modifications and expression of a large number of genes, and special AT-rich sequence-binding protein 2 (SATB2) is a close homologue with similar functions [3-5].

SATB1-expression has been demonstrated to confer a more aggressive tumour phenotype and a shorter patient survival in several cancer forms, e.g. breast cancer [3], prostate cancer [6], laryngeal squamous cell carcinoma [7], nasopharyngeal cancer [8], hepatocellular carcinoma [9], rectal cancer [10], cutaneous malignant melanoma [11], epithelial ovarian cancer [12], glioma [13] and gastric cancer [14].

The SATB2 gene is involved in osteoblast differentiation and craniofacial patterning [15,16] and has been demonstrated to be abundantly expressed in normal colorectal mucosa and colorectal adenocarcinomas, but more sparsely in other types of carcinomas [17]. Low or absent SATB2-expression has further been shown to be a marker of malignant behaviour and poor prognosis in colorectal cancer [18,19], whereas high expression correlated to a better response to neoadjuvant chemotherapy in rectal cancer and neoadjuvant/adjuvant chemotherapy in stage III-IV colorectal cancer [20].

The expression and prognostic significance of SATB1 and SATB2 in pancreatic, distal bile duct, ampullary or duodenal adenocarcinomas has not yet been reported. The aim of the present study was therefore to examine the expression, clinicopathological correlates, and prognostic and treatment predictive ability of SATB1 and SATB2 in primary tumours (n = 175) and paired lymph node metastases (n = 105) from a consecutive cohort of patients with periampullary adenocarcinoma, including pancreatic cancer.

## Methods

### Patients

The study cohort is a previously described retrospective consecutive series of 175 pancreaticoduodenectomy specimens with primary adenocarcinomas surgically treated at the University hospitals of Lund and Malmö, Sweden, from January 1 2001 until December 31 2011 [21]. Data on survival were gathered from the Swedish National Civil Register. Follow-up started at the date of surgery and ended at death, at 5 years after surgery or at December 31 2013, whichever came first. Information on neoadjuvant and adjuvant treatment and recurrence was obtained from patient records.

All haematoxylin & eosin stained slides from all cases were re-evaluated by one pathologist (JEL), blinded to the original report and outcome, with the decision on tumour origin and morphological type being based on several criteria, as previously described [21].

The study has been approved by the Ethics Committee of Lund University (ref nr 445/07).

### Tissue microarray construction

Tissue microarrays (TMAs) were constructed using a semi-automated arraying device (TMArrayer, Pathology Devices, Westminister, MD, USA). A standard set of three tissue cores (1 mm) were obtained from each of the 175 primary tumours and from lymph node metastases from 105 of the cases, whereby one to three lymph node metastases were sampled in each case.

### Immunohistochemistry and staining evaluation

For immunohistochemical analysis of SATB1 and SATB2 expression, 4 μm TMA-sections were automatically pre-treated using the PT Link system and then stained in an Autostainer Plus (DAKO; Glostrup, Copenhagen, Denmark) with anti-SATB1, clone EPR3895, Epitomics, Burlingame, CA, USA, and anti-SATB2 #AMAb90679 CL0320, Atlas Antibodies AB, Stockholm, Sweden. Expression of SATB1 and SATB2 was denoted as positive when there was nuclear positivity of any intensity in at least 1 percent of cancer cells. Cases denoted as positive in any of the TMA-cores of the primary tumour or a lymph node metastasis were considered positive. Stromal lymphocytes served as a positive control for SATB1 and normal colorectal mucosa as a positive control for SATB2.

### Statistical analysis

Chi square test was applied to analyse the relationship between SATB1 expression and clinicopathological parameters. Two patients with PB-type adenocarcinomas who had received neoadjuvant chemotherapy were excluded from the correlation and survival analyses. Three additional patients were excluded from the survival analyses; two with I-type adenocarcinomas who died within one month from surgery due to complications and one with PB-type adenocarcinoma who emigrated 5 months after surgery.

Kaplan Meier estimates of 5-year overall survival (OS) and recurrence-free survival (RFS) and log rank test were applied to evaluate survival differences in strata according to positive and negative SATB1 and SATB2 expression. Hazard ratios (HR) for death and recurrence within 5 years were calculated by Cox regression proportional hazard’s modelling in unadjusted analysis and in a multivariable model adjusted for age, sex, T-stage, N-stage, differentiation grade, lymphatic invasion, vascular invasion, perineural invasion, infiltration in peripancreatic fat, resection margins, tumour origin, and adjuvant chemotherapy. A backward conditional method was used for variable selection in the adjusted model. To estimate the interaction effect between adjuvant treatment and SATB1 expression in order to measure any possible difference in treatment effect based on SATB1 expression, the following interaction variables were constructed; any adjuvant treatment (+/−) × SATB1 (+/−), and gemcitabine-based treatment (+/−) × SATB1 (+/−).

All tests were two sided. P-values <0.05 were considered significant. All statistical analyses were performed using IBM SPSS Statistics version 20.0 (SPSS Inc., Chicago, IL, USA).

## Results

### Associations of SATB1 expression with clinicopathological factors and SATB2 expression

**Figure 1:**
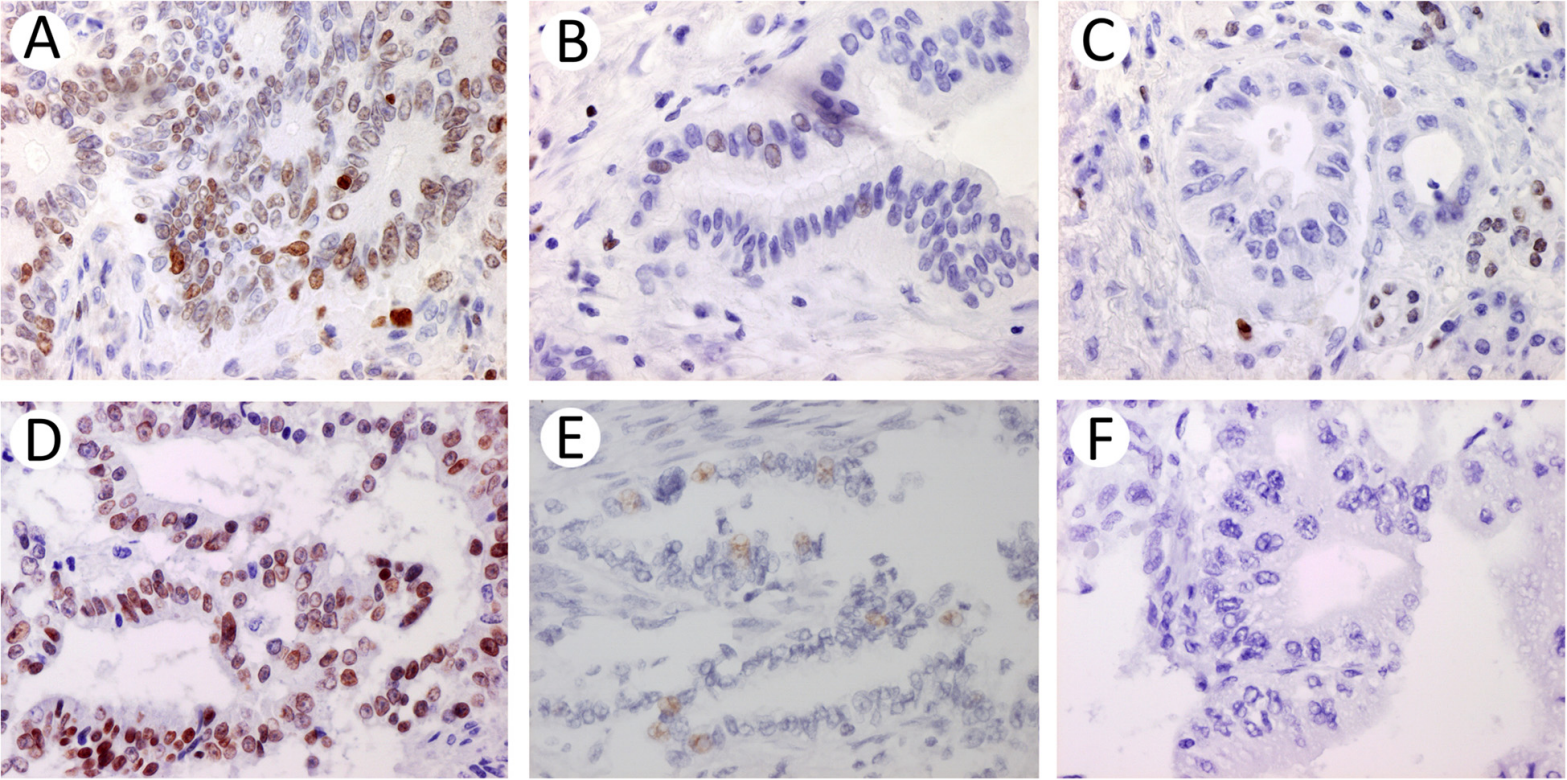
**Immunohistochemical stains of SATB1 (A-C) and SATB2 (D-F) showing varying fractions and intensities of positive cells (A-B and D-E) and negative stains (C and F). B** and **E** show low fractions of weakly positive cancer cells, in tumours denoted as positive.

Sample immunohistochemical images of SATB1 and SATB2 expression are shown in Figure 1.

In the full cohort of 175 cases there were 110 PB-type and 65 I-type adenocarcinomas. Two patients with PB-type carcinoma who had received neoadjuvant chemotherapy were excluded from the analyses. Among the remaining cases, SATB1 expression could be assessed in 106/108 (98.1%) primary PB-type carcinomas; 16 (15.1%) being denoted as positive and 90 (84.9%) as negative, and in 65/75 (86.7%) metastases; 11(16.9%) being denoted as positive and 54 (83.1%) as negative. Out of the 11 cases with positive SATB1 expression in a metastasis, 6 (54.5%) had positive and 5 (45.5%) had negative SATB1 expression in the corresponding primary tumour. Using a combined variable wherein SATB1 expression of any intensity in >1% cells in the primary tumour and/or metastases was denoted as positive, 21 (19.8%) PB-type cases had positive and 85 (80.2) cases had negative SATB1-expression (Table 1). SATB1 was assessable in 63/65 (96.9%) primary I-type carcinomas; 15 (23.8%) being positive and 48 (76.2%) being negative, and in 26/30 (86.7%) metastases; 4 (15.4%) being positive and 22 (84.6%) being negative. Out of the 4 cases with positive expression in a metastasis, 3 (75%) also displayed positive expression in the corresponding primary tumour. When combining positivity in primary tumours and/or metastases, there were 16 (25.4%) SATB1 positive and 47 (74.6%) negative I-type cases (Table 1).

**Table 1:** **SATB1-expression in relation to clinicopathological parameters and SATB2-expression**

	**Pancreatobiliary type**				**Intestinal type**			
	**SATB1 - n = 85**	**SATB1 + n = 21**	**SATB 1 missing n = 2**	**p-value**	**SATB1- n = 47**	**SATB1+ n = 16**	**SATB1 missing n = 2**	**p-value**
Age, years, M (IQR)	66 (61–72)	69 (64–74)	2	0.681	67 (62–72)	67 (57–70)	2	0.981
Sex, n (%)				0.469				0.777
Women	41 (48%)	8 (38%)	2		26 (55%)	8 (50%)	1	
Men	44 (52%)	13 (62%)			21 (45%)	8 (50%)	1	
Tumour origin, n (%)				0.852				0.487
Duodenum					12 (26%)	2 (13%)		
Ampulla Intestinal type					35 (74%)	14 (87%)	2	
Ampulla Pancreatobiliary type	16 (19%)	3 (14%)						
Distal bile duct	34 (40%)	10 (48%)	1					
Pancreas	35 (41%)	8 (38%)	1					
Tumour size, mm, M (IQR)	30 (25–35)	28 (21–30)	2	0.799	25 (15–40)	30 (24–40)	2	0.848
Differentiation grade, n (%)				0.211				0.148
Well-moderate	34 (40%)	5 (24%)	1		26 (55%)	5 (31%)	1	
Poor	51 (60%)	16 (76%)	1		21 (45%)	11 (69%)	1	
T-stage, n (%)				1.000				0.860
T1	2 (2%)	0	1		4 (9%)	0	1	
T2	8 (9%)	2 (10%)			8 (17%)	3 (19%)	1	
T3	61 (72%)	16 (76%)	1		18 (38%)	7 (44%)		
T4	14 (16%)	3 (14%)			17 (36%)	6 (37%)		
N-stage, n (%)				0.421				0.564
N0	25 (29%)	4 (19%)	2		26 (55%)	7 (44%)	2	
N1-N2	60 (71%)	17 (81%)			21 (45%)	9 (56%)		
Margins, n (%)				1.000				1.000
R0	5 (6%)	1 (5%)	1		13 (28%)	4 (25%)	1	
R1-Rx	80 (94%)	20 (95%)	1		34 (72%)	12 (75%)	1	
Perineural growth, n (%)				0.232				0.533
No	20 (24%)	2 (10%)	1		34 (72%)	10 (62%)	1	
Yes	65 (76%)	19 (90%)	1		13 (28%)	6 (38%)	1	
Invasion of lymphatic vessels, n (%)				0.792				0.081
No	25 (29%)	7 (33%)	1		25 (53%)	4 (25%)		
Yes	60 (71%)	14 (67%)	1		22 (47%)	12 (75%)	2	
Invasion of blood vessels, n (%)				0.070				0.594
No	60 (71%)	10 (48%)	1		44 (94%)	14 (87%)	2	
Yes	25 (29%)	11 (52%)	1		3 (6%)	2 (13%)		
Growth in peripancreatic fat, n (%)				0.760				0.545
No	18 (21%)	3 (14%)	2		32 (68%)	9 (56%)	2	
Yes	67 (79%)	18 (86%)			15 (32%)	7 (44%)		
SATB2, n (%)				0.092				0.422
Negative	84 (99%)	18 (90%)	2		40 (89%)	13 (81%)	0	
Positive	1 (1%)	2 (10%)	0		5 (11%)	3 (19%)	0	
Missing	0	1	0		2	0	2	
Adjuvant chemotherapy, n (%)				0.739				0.301
No adjuvant	41 (48%)	9 (43%)	1		35 (74%)	10 (63%)	2	
5FU-analogue	5 (6%)	3 (14%)			4 (9%)	1 (6%)		
Gemcitabine	35 (41%)	9 (43%)			5 (11%)	2 (13%)		
Gemcitabine + capecitabine	1 (1%)	0	1		0	1 (6%)		
Oxaliplatin +5-FU analogue	1 (1%)	0			3 (6%)	1 (6%)		
Gemcitabine + oxaliplatin	2 (2%)	0			0	1 (6%)		
Recurrence				0.250				0.658
No	16 (19%)	3 (14%)	1		27 (57%)	7 (44%)	1	
Yes, local only	25 (29%)	3 (14%)	1		3 (6%)	1 (6%)		
Yes, non-local	44 (52%)	15 (71%)			17 (36%)	8 (50%)	1	
Included in survival analyses				1.000				1.000
Yes	84 (99%)	21 (100%)	0		45 (96%)	16 (100%)	0	
No	1 (1%)	0	2		2 (4%)	0	2	

There were no significant associations between SATB1-expression, clinicopathological characteristics and SATB2-expression.

There were no significant associations between SATB1-expression and clinicopathological parameters (Table 1). Among SATB1-positive PB-cases there was a tendency towards a higher proportion of cases with blood vessel involvement (p = 0.070), compared with SATB1-negative cases. Among SATB1-positive I-type cases there was a tendency towards a higher proportion of cases with lymphatic vessel involvement (p = 0.081), compared with SATB1-negative cases.

SATB2 expression was assessable in 107/108 (99.1%) PB-type primary tumours, and denoted as positive in 3 (2.8%) cases and negative in 104 (97.2%) cases. There were 2 positive PB-type metastases, both corresponding to positive primary tumours. Among 61/65 (93.8%) assessable I-type primary tumours SATB2 was positive in 8 (13.1%), and negative in 53 (86.9%) cases. There were 3 positive I-type metastases, all corresponding to positive primary tumours.

SATB1 expression was positive in 2 and negative in 1 of the 3 cases with SATB2-positive PB-type tumours. Three of the 8 SATB2-positive I-type cases were SATB1-positive, and 5 were negative. There were no significant associations between SATB1 and SATB2 expression in either of the morphological groups (Table 1).

SATB2 expression was significantly associated with growth in peripancreatic fat in I-type tumours (p = 0.042), but not with any other clinicopathological factor, and there were no significant associations in PB-type tumours (Additional file 1: Table S1).

There was a significant association between gemcitabine based adjuvant chemotherapy and tumour origin in PB-type tumours, and between adjuvant chemotherapy and involved lymph nodes in intestinal type tumours (Table 2). Except for these two factors, the distribution of patient and tumour characteristics did not differ significantly between patients who had received or not received adjuvant chemotherapy in neither of the histological subtypes.

**Table 2:** **Adjuvant chemotherapy in relation to clinicopathological parameters**

	**Pancreatobiliary type**			**Intestinal type**		
	**No adjuvant or non-gemcitabine based n = 60**	**Gemcitabine based n = 50**	**p-value**	**No adjuvant n = 47**	**Any adjuvant n = 18**	**P-value**
No follow up, n	1	0	1.000	2	0	1.000
Received neoadjuvant treatment, n	0	2	0.204	0	0	
Sex			0.253			1.000
Female, n (%)	31 (61%)	20 (39%)		25 (71%)	10 (29%)	
Male, n (%)	29 (49%)	30 (51%)		22 (73%)	8 (27%)	
Age at surgery, years. M (IQR)	69 (62–73)	66 (60–70)	0.260	67 (62–72)	67 (56–71)	0.441
Tumour origin			**0.002**			0.316
Pancreas, n (%)	16 (35%)	30 (65%)				
Distal bile duct, n (%)	30 (67%)	15 (33%)				
Ampulla of Vater, n (%)	14 (74%)	5 (26%)		35 (69%)	16 (31%)	
Duodenum, n (%)				12 (86%)	2 (14%)	
Tumour size, mm. M (IQR)	30 (22–37)	30 (25–35)	0.702	23 (13–40)	30 (24.5-40)	0.690
Tumour grade			0.555			0.783
Well/moderate, n (%)	21 (50%)	21 (50%)		24 (75%)	8 (25%)	
Poor, n (%)	39 (57%)	29 (43%)		23 (70%)	10 (30%)	
Lymph nodes			0.531			**0.013**
Uninvolved (N0), n (%)	20 (61%)	13 (39%)		30 (86%)	5 (14%)	
Involved (N1-N2), n (%)	40 (52%)	37 (48%)		17 (57%)	13 (43%)	
Margins			0.452			0.230
Uninvolved, n (%)	5 (71%)	2 (29%)		11 (61%)	7 (39%)	
Involved or unknown, n (%)	55 (53%)	48 (47%)		36 (77%)	11 (23%)	
Perineural growth			0.362			0.229
No, n (%)	16 (64%)	9 (36%)		35 (78%)	10 (22%)	
Yes, n (%)	44 (52%)	41 (48%)		12 (60%)	8 (40%)	
Growth in lymph vessels			0.223			1.000
No, n (%)	16 (46%)	19 (54%)		21 (72%)	8 (28%)	
Yes, n (%)	44 (59%)	31 (41%)		26 (72%)	10 (28%)	
Growth in blood vessels			0.312			1.000
No, n (%)	37 (51%)	36 (49%)		43 (72%)	17 (28%)	
Yes, n (%)	23 (62%)	14 (38%)		4 (80%)	1 (20%)	
Growth in peripancreatic fat			0.649			0.142
No, n (%)	15 (60%)	10 (40%)		34 (79%)	9 (21%)	
Yes, n (%)	45 (53%)	40 (47%)		13 (59%)	9 (41%)	
T-stage			0.240			0.301
T1, n (%)	2 (67%)	1 (33%)		5 (100%)	0	
T2, n (%)	4 (33%)	8 (67%)		10 (83%)	2 (17%)	
T3, n (%)	42 (54%)	36 (46%)		18 (72%)	7 (28%)	
T4, n (%)	12 (71%)	5 (29%)		14 (61%)	9 (39%)	
Year of surgery. M (IQR)	2007.5 (2004–2010)	2009 (2007–2010)	**0.004**	2006 (2003–2009)	2009 (2006.5-2010)	0.372

M, median. IQR, interquartile range. Bold text indicates significant p-values.

### Prognostic and treatment predictive value of SATB1 expression in pancreatobiliary type tumours

**Figure 2:**
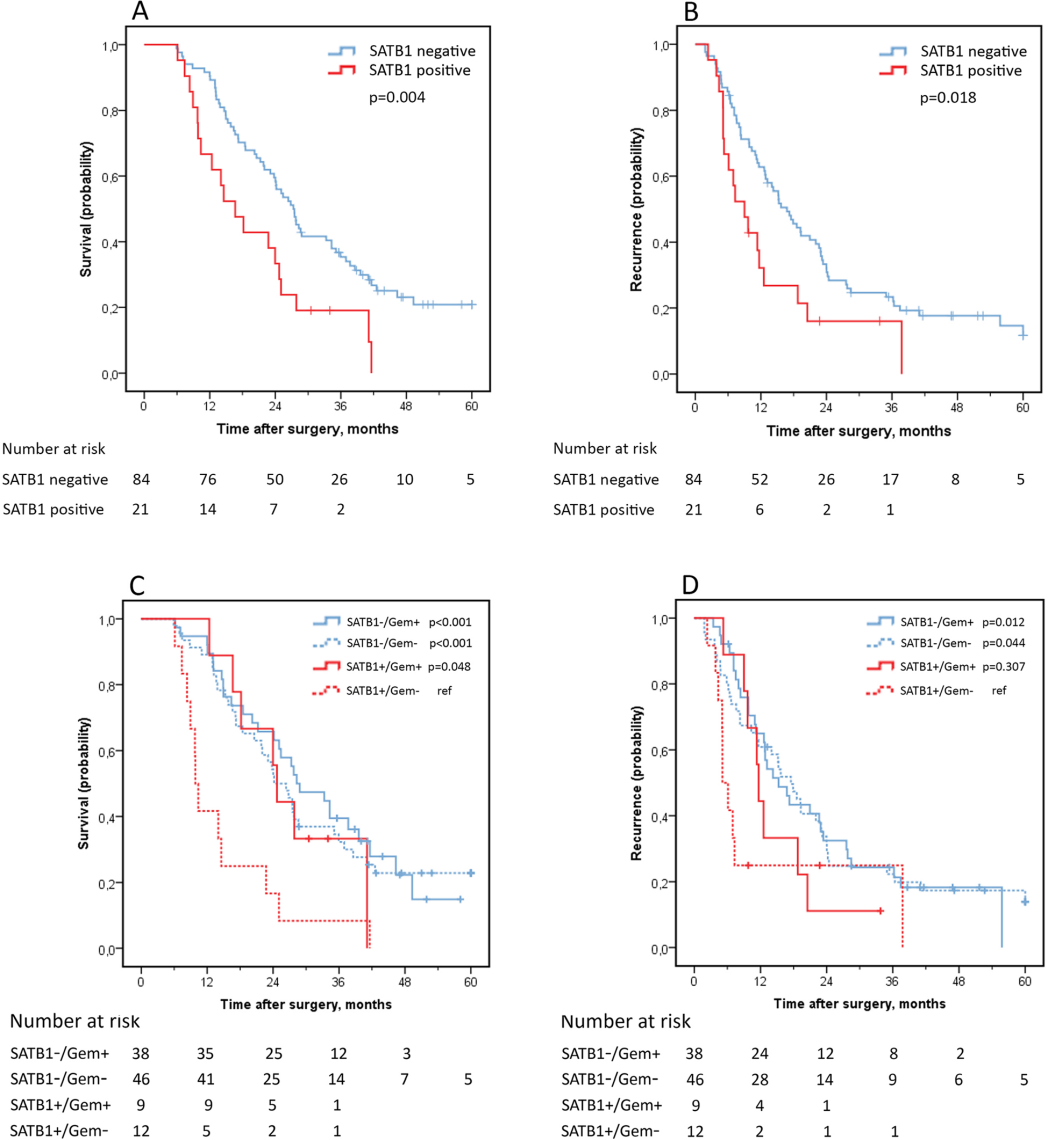
**Kaplan-Meier estimates of overall survival (A) and recurrence free survival (B) in pancreatobiliary type tumours stratified by SATB1-expression and corresponding curves stratified for adjuvant chemotherapy (C-D).**

As demonstrated in Figure 2A-B, Kaplan-Meier analysis revealed that SATB1 expression was prognostic for OS and RFS in the PB-group of tumours. SATB1 positive cases had a shorter OS compared with SATB1 negative cases, median 16.7 months (interquartile range, IQR 9.9-25.1) vs 27.3 months (IQR 15.8-46.3) (logrank p = 0.004), and also a shorter RFS, median 9.0 months (IQR 5.1-18.8) vs 16.8 months (IQR 8.0-28.5) (logrank p = 0.018). As demonstrated in Table 3, the significant associations of SATB1 expression with survival were confirmed in Cox univariable analysis for both OS (HR = 2.11; 95% confidence interval, CI 1.25-3.56) and RFS (HR = 1.87; 95% CI 1.10-3.18), and this significance was retained for OS in multivariable analysis (HR = 1.79; 95% CI 1.05-3.05).

**Table 3:** **Hazard ratios for overall survival and recurrence free survival in pancreatobiliary type tumours**

	**Pancreatobiliary type**			
	**OS**		**RFS**	
	**Univariable**	**Multivariable**	**Univariable**	**Multivariable**
Age	0.99 (0.96-1.02)	1.02 (0.99-1.05)	0.98 (0.96-1.01)	0.99 (0.96-1.02)
Sex				
Women				
Men	1.24 (0.80-1.91)	1.02 (0.64-1.64)	1.09 (0.72-1.66)	0.82 (0.52-1.30)
Tumour size				
	**1.03 (1.01-1.05)**	1.01 (0.99-1.04)	**1.04 (1.02-1.05)**	1.01 (0.99-1.04)
Tumour grade				
Well-moderate				
Poor	**2.50 (1.54-4.05)**	**2.10 (1.28-3.45)**	**2.40 (1.50-3.83)**	**2.35 (1.43-3.84)**
Tumour origin				
Ampulla				
Distal bile duct	0.74 (0.40-1.34)	1.02 (0.53-1.98)	1.10 (0.61-1.97)	2.68 (0.33-21.81)
Pancreas	0.88 (0.49-1.60)	1.08 (0.56-2.08)	1.01 (0.56-1.84)	2.26 (0.27-18.81)
T-stage				
T1				
T2	1.93 (0.23-16,04)	0.54 (0.06-5.05)	2.21 (0.27-18.38)	0.61 (0.06-5.75)
T3	3.99 (0.55-28.85)	0.74 (0.09-6.10)	6.43 (0.89-46.43)	1.28 (0.16-10.29)
T4	5.11 (0.67-38.79)	2.36 (0.11-49.41)	5.95 (0.78-45.43)	0.93 (0.11-8.00)
N-stage				
N0				
N1	**2.55 (1.49-4.38)**	**2.49 (1.42-4.38)**	**2.59 (1.55-4.33)**	**2.15 (1.22-3.80)**
Margin status				
R0				
R1-Rx	4.02 (0.99-16.38)	2.43 (0.59-10.02)	2.71 (0.99-7.44)	2.30 (0.82-6.50)
Perineural				
Pn0				
Pn1	**1.97 (1.10-3.53)**	1.04 (0.50-2.15)	**3.09 (1.66-5.75)**	1.80 (0.94-3.46)
Lymphatic vessels				
L0				
L1	1.57 (0.96-2.56)	1.02 (0.57-1.85)	**1.85 (1.14-3.01)**	1.14 (0.65-2.00)
Blood vessels				
V0				
V1	**2.43 (1.56-3.78)**	**2.53 (1.59-4.03)**	**2.35 (1.50-3.69)**	**1.96 (1.21-3.17)**
Peripancreatic fat				
Pn0				
Pn1	**1.89 (1.05-3.40)**	0.94 (0.47-1.90)	**2.75 (1.50-5.02)**	1.78 (0.94-3.40)
SATB1				
Negative				
Positive	**2.11 (1.25-3.56)**	**1.79 (1.05-3.05)**	**1.87 (1.10-3.18)**	1.54 (0.89-2.66)
Adjuvant chemotherapy				
None/other				
Gemcitabine	0.76 (0.49-1.18)	**0.56 (0.35-0.89)**	0.98 (0.64-1.49)	0.72 (0.46-1.12)

Bold text indicates significant values.

SATB1-positive cases receiving adjuvant gemcitabine had a prolonged OS, median 24.7 (IQR 18.2-41.1), compared with SATB1-positive cases not receiving adjuvant gemcitabine, median 9.9 (IQR 8.3-14.6) (logrank p = 0.048, Figure 2C), while there was no significant difference in OS between SATB1-negative cases receiving (38/84) or not receiving (46/84) adjuvant gemcitabine (Figure 2C). The interaction between SATB1 and adjuvant gemcitabine in relation to OS approached significance, p(interaction) = 0.066 (Table 4).

**Table 4:** **Cox proportional hazards analysis of the impact of SATB1 protein expression on overall survival and recurrence free survival in resected pancreatobiliary type and intestinal type periampullary adenocarcinomas**

	**OS**			**RFS**		
**Pancreatobiliary type**	**HR (95% CI)**	**n (events)**	**p†**	**HR (95% CI)**	**n (events)**	**p** ^**†**^
**All cases**						
SATB1 neg	1.00	84 (63)		1.00	84 (69)	
SATB1 pos	**2.11 (1.25-3.56)**	21 (19)		**1.87 (1.10-3.18)**	21 (18)	
**No adjuvant treatment**			0.166			0.927
SATB1 neg	1.00	40 (30)		1.00	40 (33)	
SATB1 pos	**2.94 (1.37-6.29)**	9 (9)		1.63 (0.71-3.74)	9 (7)	
**Any adjuvant treatment**						
SATB1 neg	1.00	44 (33)		1.00	44 (36)	
SATB1 pos	1.70 (0.83-3.52)	12 (10)		**2.05 (1.02-4.11)**	12 (11)	
**No gemcitabine**			0.066			0.384
SATB1 neg	1.00	46 (35)		1.00	46 (38)	
SATB1 pos	**3.14 (1.60-6.16)**	12 (12)		**2.05 (1.00-4.20)**	12 (10)	
**Gemcitabine**						
SATB1 neg	1.00	38 (28)		1.00	38 (31)	
SATB1 pos	1.44 (0.62-3.35)	9 (7)		1.60 (0.72-3.56)	9 (8)	
**Intestinal type**						
**All cases**						
SATB1 neg	1.00	45 (22)		1.00	45 (20)	
SATB1 pos	1.06 (0.47-2.38)	16 (8)		1.26 (0.57-2.77)	16 (9)	
**No adjuvant treatment**			0.165			**0.021**
SATB1 neg	1.00	33 (17)		1.00	33 (13)	
SATB1 pos	1.62 (0.67-3.92)	10 (7)		**2.69 (1.11-6.51)**	10 (8)	
**Any adjuvant treatment**						
SATB1 neg	1.00	12 (5)		1.00	12 (7)	
SATB1 pos	0.30 (0.03-2.56)	6 (1)		0.18 (0.02-1.46)	6 (1)	
**No gemcitabine**			0.649			0.143
SATB1 neg	1.00	40 (20)		1.00	40 (17)	
SATB1 pos	1.20 (0.51-2.83)	12 (7)		1.76 (0.76-4.09)	12 (8)	
**Gemcitabine**						
SATB1 neg	1.00	5 (2)		1.00	5 (3)	
SATB1 pos	0.67 (0.06-7.53)	4 (1)		0.27 (0.03-2.64)	4 (1)	

^†^P value for term of interaction by Cox multivariable analysis including treatment, SATB1 expression, gemcitabine vs no gemcitabine or any adjuvant vs no adjuvant, and a term of interaction. Bold text indicates significant values.

Similar findings were obtained when considering SATB1 expression in primary tumours only; with a significantly shorter OS for SATB1 positive PB-cases (logrank p = 0.021) and a difference in response to adjuvant gemcitabine in SATB1 positive cases (8/16 receiving vs 8/16 not receiving adjuvant gemcitabine, logrank p = 0.054) compared with negative cases (39/89 receiving vs 50/89 not receiving adjuvant gemcitabine, logrank p = 0.491) and p(interaction) =0.067.

### Prognostic and treatment predictive value of SATB1 expression in intestinal type tumours

**Figure 3:**
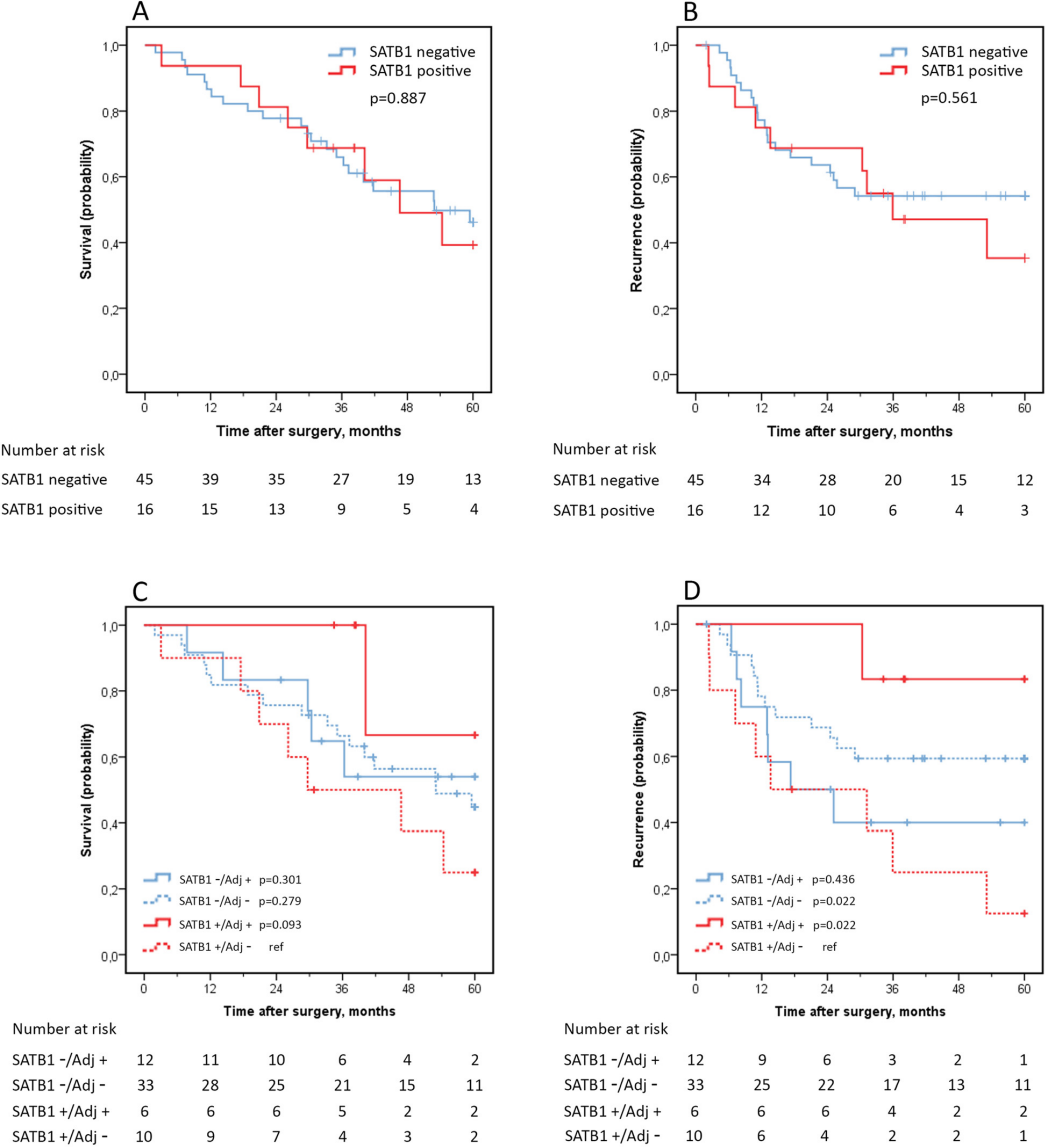
**Kaplan-Meier estimates of overall survival (A) and recurrence free survival (B) in intestinal type tumours stratified by SATB1-expression and corresponding curves stratified for adjuvant chemotherapy (C-D).**

In contrast to the PB-group, SATB1 expression was not prognostic for OS or RFS in the I-type category of tumours (Figure 3A-B). However, while there was no significant difference in OS or RFS between SATB1-negative cases receiving (12/45) or not receiving (33/45) adjuvant chemotherapy (logrank p = 0.866), there was a tendency towards a prolonged OS for cases with SATB1-positive tumours receiving adjuvant chemotherapy (6/16), median n.r. (IQR 40.2-n.r.), compared with SATB1-positive cases not receiving adjuvant chemotherapy (10/16), median 29.7 (IQR 20.9-54.3) (logrank p = 0.093) (Figure 3C). SATB1-positive cases receiving adjuvant chemotherapy (6/16) also had a prolonged RFS, median n.r. (IQR n.r-n.r.), compared with SATB1-positive cases not receiving adjuvant chemotherapy (10/16), median 13.6 (IQR 7.2-35.9) (logrank p = 0.022) and there was a tendency towards a prolonged RFS in SATB1-positive cases receiving adjuvant chemotherapy compared to SATB1-negative cases receiving adjuvant chemotherapy (logrank p = 0.071). There was no significant difference in RFS between SATB1-negative cases receiving (12/45) or not receiving adjuvant chemotherapy (33/45) (logrank p = 0.257) (Figure 3D). There was a significant interaction between SATB1 and adjuvant chemotherapy in relation to RFS in I-type tumours, p(interaction) = 0.021.

Similar results were seen when considering SATB1 expression in primary I-type tumours only; no difference in RFS between SATB1-negative cases receiving or not receiving adjuvant chemotherapy (logrank p = 0.332) while RFS differed significantly between SATB1-positive cases receiving or not receiving adjuvant chemotherapy (logrank p = 0.031). The interaction between SATB1 and adjuvant chemotherapy in relation to RFS was significant also when considering positivity in primary tumours only, p(interaction) = 0.032.

### Prognostic and treatment predictive value of SATB2 expression

**Figure 4:**
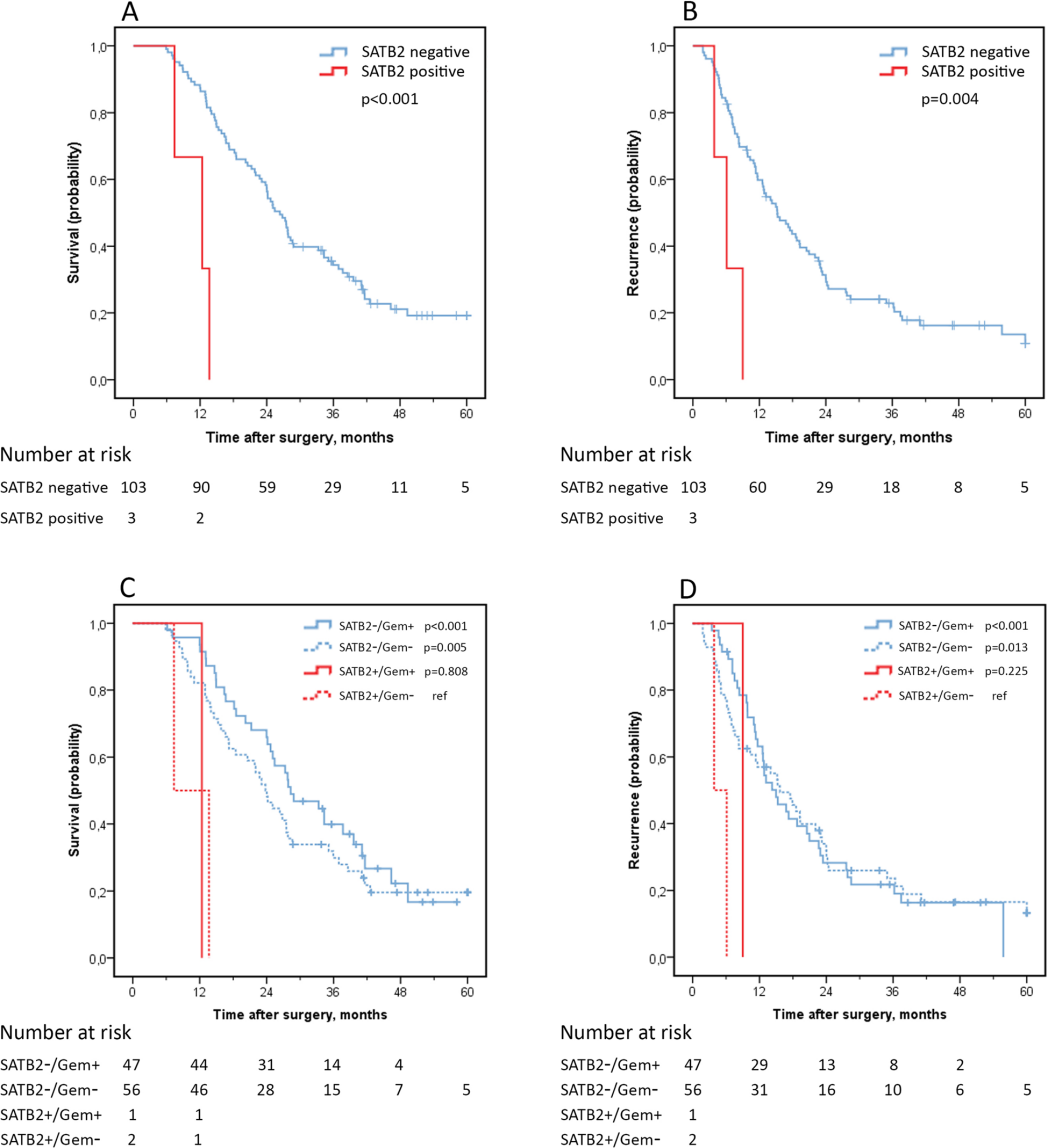
**Kaplan-Meier estimates of overall survival (A) and recurrence free survival (B) in pancreatobiliary type tumours stratified by SATB2-expression and corresponding curves stratified for adjuvant chemotherapy (C-D).**

SATB2-expression was only seen in 3 out of 107 PB-type tumours, making the statistical analyses hazardous to interpret. However, as demonstrated in Figure 4A-B, a significantly shorter OS and RFS was observed for the small number of cases having SATB2-positive tumours, and this significance was retained in both univariable analysis for OS and RFS (HR 7.79; 95% CI 2.29-26.51 and HR 4.93; 95% CI 1.50-16.2) and in multivariable analysis for OS and RFS (HR 4.08; 95% CI 1.18-14.11 and HR 6.40; 95% CI 1.90-21.58).

**Figure 5:**
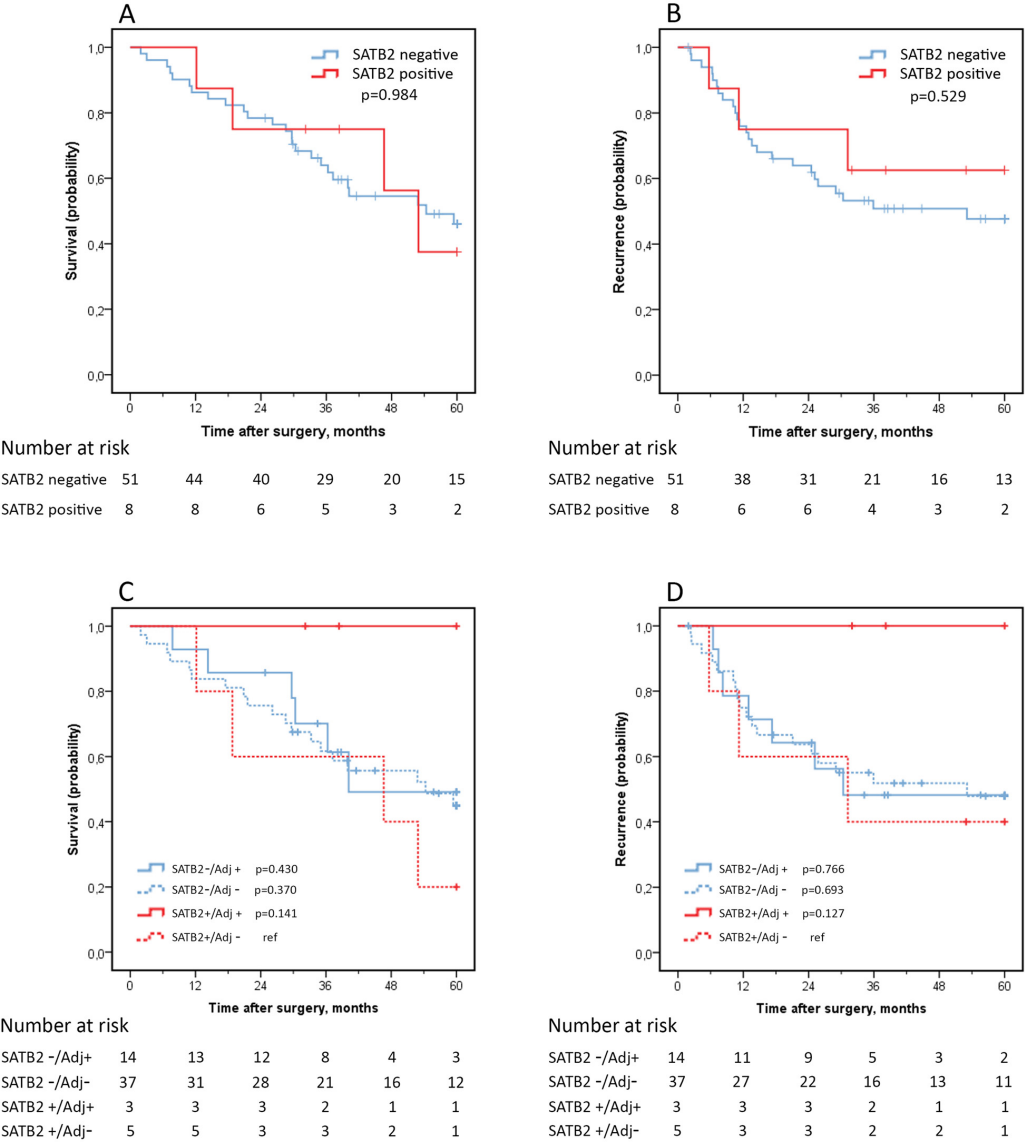
**Kaplan-Meier estimates of overall survival (A) and recurrence free survival (B) in intestinal type tumours stratified by SATB2-expression and corresponding curves stratified for adjuvant chemotherapy (C-D).**

In I-type tumours, SATB2-positivity was seen in 8 out of 61 cases. Expression of SATB2 was however not prognostic, for OS or RFS (Figure 5A-B). Moreover, there were no significant differences in survival between SATB2-positive cases receiving or not receiving adjuvant chemotherapy, but, of note, there were no recurrences or fatalities among SATB2-positive I-type cases receiving adjuvant chemotherapy (Figure 5C-D).

## Discussion

The results from this study provide a first demonstration of the expression and prognostic value of SATB1 in pancreatic, distal bile duct, ampullary and duodenal adenocarcinoma. Positive SATB1-expression was observed in 20% of resected PB-type cases, and was associated with a shorter RFS and OS, which is in line with previous publications on the prognostic significance of SATB1 expression in several other major types of cancer [3,7,9-12,14]. The findings from the present study thus provide further evidence of SATB1 being a master regulator towards a more aggressive tumour phenotype and a biomarker of poor prognosis in human cancer. In addition, the finding of a potential treatment predictive role of SATB1, its expression being associated with a better response to adjuvant gemcitabine in PB-type tumours, reflected in a prolonged 5-year survival, and an improved response to any adjuvant chemotherapy in I-type tumours, reflected in a prolonged recurrence-free survival, has however not yet been described in any type of cancer. Patients with pancreatic and periampullary adenocarcinomas have a very dismal prognosis even after surgical removal of the tumour. According to contemporary treatment protocols, all patients with pancreatobiliary type adenocarcinoma, including pancreatic cancer are recommended adjuvant treatment, and adjuvant chemotherapy with gemcitabine has recently been shown to increase overall and disease-free survival among patients with radically resected tumours [22]. A challenging task is however to identify which patients will actually benefit from this treatment and not only suffer from the adverse side effects resulting in a reduced quality of life. The here examined retrospective cohort consists of a comparatively large proportion of patients who did not receive any adjuvant chemotherapy, which is in part likely due to the fact that all types of periampullary adenocarcinomas are included. As shown in Table 2, tumour origin and year of surgery differs between the gemcitabine and non-gemcitabine groups of PB-type tumours. During the first part of the included period (2001–2011), the distinction between pancreatobiliary and intestinal tumour morphology was not made, and decision on adjuvant chemotherapy seems to have been based mainly on tumour origin. Many PB-type ampullary tumours did thus not receive adjuvant chemotherapy and tumours of distal bile duct origin were given adjuvant chemotherapy less often than tumours of pancreatic origin. For intestinal type tumours, decision on adjuvant chemotherapy seems to have been based primarily on involved lymph nodes, as this is the only parameter that differs significantly between the group that received and those that did not receive adjuvant chemotherapy. Although treatment predictive effects are best studied in a randomized setting, the nearly equal distribution of patients treated or not treated with adjuvant chemotherapy in this retrospective cohort provides a better setting for discovery of potential treatment predictive markers than studies on cohorts where all patients have received adjuvant chemotherapy.

Apart from considerations in the adjuvant situation, SATB1 could also prove to be a useful biomarker for identification of patients with borderline resectable tumours who will respond well to neoadjuvant chemotherapy, thus increasing the number of resectable tumours. Therefore, the indication of a treatment predictive value of SATB1 expression in periampullary adenocarcinoma is of high potential clinical relevance and merits further validation in additional patient cohorts. The mechanistic basis for SATB1-related increased sensitivity to various combinations of chemotherapy should also be pursued in future studies.

Given the high homology of SATB1 and SATB2, it is important to use well-validated antibodies to ensure target specificity. The antibodies used in the present study have been validated previously [23] and cross-reactivity should therefore not be an issue.

SATB1 was often heterogeneously expressed and the number of positive cells was often low, which justifies assessment not only of the primary tumour, but also metastases in order to improve the detection of positive cases.

Immunohistochemistry has several advantages compared to e.g. analyses of mRNA levels in that it allows for assessment of candidate protein biomarkers in a morphological and subcellular context. The results from this study are quite in line with several previous studies demonstrating that even a small fraction of SATB1 positive cells by immunohistochemistry is sufficient to confer a poor prognosis [3,12]. Moreover, results from studies on the prognostic value of mRNA levels of SATB1 have shown discrepant results in relation to its protein expression in e.g. breast cancer [24,25]. A likely explanation for this is the more or less abundant expression of SATB1 in activated lymphocytes, also serving as a positive internal control in immunohistochemical studies. Therefore, immunohistochemistry should be the method of choice for assessment of the utility of SATB1 as a prognostic and treatment predictive biomarker in human cancer.

Some methodological aspects on the TMA technique need consideration. Although heterogeneity issues cannot be fully circumvented, it is reasonable to assume that analysis of whole tissue sections will lead to an improved detection rate of positive primary tumours and/or metastases. However, while the use of whole sections is feasible in the clinical setting and in prospective studies, the TMA technique has become a well-established platform for high-throughput tissue biomarker studies in the retrospective setting, and has been demonstrated to provide similar or even better prognostic information for heterogeneously expressed markers than whole section-based analyses [26]. Moreover, a comparative strength of the here used TMA is that tissue cores had, whenever possible, been obtained from different donor blocks of the primary tumours, and from different lymph node metastases in cases with more than one metastasis.

In a previous study related to the potential utility of SATB2 as a diagnostic marker for colorectal cancer, screening of its expression in a multitude of normal and cancerous tissues revealed that none out of 25 pancreatic adenocarcinomas and only one out of 15 bile duct adenocarcinomas were positive for SATB2 [17], which is in line with the finding in the present study of SATB2 being positive in only three out of 107 pancreatobiliary type adenocarcinomas. Although a significant association was found between SATB2 expression and poor prognosis, the small number of positive cases makes it hazardous to draw any conclusions on the potential prognostic value of SATB2 expression in PB-type tumours. In the group of I-type tumours, where SATB2 expression was more frequent (8/61), no prognostic effect was seen. Moreover, while there were no significant treatment predictive effects of SATB2 expression in I-type adenocarcinoma, it is noteworthy that SATB2-positive I-type cases receiving adjuvant chemotherapy had no recurrences or fatalities during the follow up period. This observation may suggest a similar treatment predictive function for SATB2 in I-type tumours as observed for SATB1 and would also be in line with the previously described treatment predictive function of SATB2 in colorectal adenocarcinoma [20]. Along this line, while some studies have suggested antagonistic effects of SATB1 and SATB2 [5,18,23], it cannot be ruled out that SATB1 and SATB2 both increase chemotherapy sensitivity in the here examined types of cancer.

## Conclusions

Expression of SATB1 is associated with poor prognosis in pancreatobiliary type adenocarcinomas, and predicts response to adjuvant treatment in both intestinal type and pancreatobiliary type periampullary adenocarcinomas, including pancreatic cancer. These findings are of potential clinical relevance and merit further validation in additional patient cohorts as well as in a mechanistic context.

## Additional file

Additional file 1: Table S1. SATB2-expression in relation to clinicopathological parameters and SATB1-expression.

## Abbreviations

SATB1: Special AT-rich sequence-binding protein 1
SATB2: Special AT-rich sequence-binding protein 2
PB-type: Pancreatobiliary type adenocarcinoma
I-type: Intestinal type adenocarcinoma
TMA: Tissue microarray
OS: Overall survival
RFS: Recurrence free survival
HR: Hazard ratio
CI: Confidence interval
IQR: Interquartile range
N.r.: Not reached
