# Supplementary material for: Prognostic and treatment predictive significance of SATB1 and SATB2 expression in pancreatic and periampullary adenocarcinoma
Source: J Transl Med. 2014 Oct 17;12:289. doi: 10.1186/s12967-014-0289-8 (PMC4232660; doi:10.1186/s12967-014-0289-8)
Supplement: Additional file 1: Table S1. — SATB2-expression in relation to clinicopathological parameters and SATB1-expression. [file 12967_2014_289_MOESM1_ESM.doc]

**Supplementary table 1. SATB2-expression in relation to clinicopathological parameters and SATB1-expression.**

|  |  | **Pancreatobiliary type** | | | | **Intestinal type** | | | |
| --- | --- | --- | --- | --- | --- | --- | --- | --- | --- |
|  |  | **SATB2- n=104** | **SATB2+ n=3** | **SATB2 missing n=1** | **p-value** | **SATB2- n=53** | **SATB2+ n=8** | **SATB2 missing n=4** | **p-value** |
| Age, years, M (IQR) | | 67 (62-73) | 69 | 1 | 0.848 | 66 (59-71) | 68 (61-69) | 4 | 0.950 |
|  |  |  |  |  |  |  |  |  |  |
| Sex, n (%) | |  |  |  | 0.246 |  |  |  | 0.283 |
|  | Women | 50 (48%) | 0 | 1 |  | 28 (53%) | 6 (75%) | 1 |  |
|  | Men | 54 (52%) | 3 (100%) | 0 |  | 25 (47%) | 2 (25%) | 3 |  |
| Tumour origin, n (%) | |  |  |  | 0.580 |  |  |  | 0.668 |
|  | Duodenum |  |  |  |  | 13 (25%) | 1 (13%) | 0 |  |
|  | Ampulla Intestinal type |  |  |  |  | 40 (75%) | 7 (87%) | 4 |  |
|  | Ampulla Pancreatobiliary type | 18 (17%) | 1 (33%) | 0 |  |  |  |  |  |
|  | Distal bile duct | 44 (42%) | 1 (33%) | 0 |  |  |  |  |  |
|  | Pancreas | 42 (41%) | 1 (33%) | 1 |  |  |  |  |  |
| Tumour size, mm, M (IQR) | | 30 (23-35) | 25 | 1 | 0.437 | 30 (18-40) | 18 (11-48) | 4 | 0.363 |
|  |  |  |  |  |  |  |  |  |  |
| Differentiation grade, n (%) | |  |  |  | 1.000 |  |  |  | 0.260 |
|  | Well-moderate | 39 (38%) | 1 (33%) | 0 |  | 27 (51%) | 2 (25%) | 3 |  |
|  | Poor | 65 (62%) | 2 (67%) | 1 |  | 26 (49%) | 6 (75%) | 1 |  |
| T-stage, n (%) | |  |  |  | 0.631 |  |  |  | 0.177 |
|  | T1 | 3 (3%) | 0 | 0 |  | 2 (4%) | 1 (12.5%) | 2 |  |
|  | T2 | 10 (10%) | 0 | 0 |  | 10 (19%) | 1 (12.5%) | 1 |  |
|  | T3 | 75 (72%) | 2 (67%) | 1 |  | 19 (36%) | 5 (62.5%) | 1 |  |
|  | T4 | 16 (15%) | 1 (33%) | 0 |  | 22 (41%) | 1 (12.5%) | 0 |  |
| N-stage, n (%) | |  |  |  | 1.000 |  |  |  | 1.000 |
|  | N0 | 30 (29%) | 1 (33%) | 0 |  | 28 (53%) | 4 (50%) | 3 |  |
|  | N1-N2 | 74 (71%) | 2 (67%) | 1 |  | 25 (47%) | 4 (50%) | 1 |  |
| Margins, n (%) | |  |  |  | 1.000 |  |  |  | 1.000 |
|  | R0 | 7 (7%) | 0 | 0 |  | 15 (28%) | 2 (25%) | 1 |  |
|  | R1-Rx | 97 (93%) | 3 (100%) | 1 |  | 38 (72%) | 6 (75%) | 3 |  |
| Perineural growth, n (%) | |  |  |  | 0.520 |  |  |  | 1.000 |
|  | No | 22 (21%) | 1 (33%) | 0 |  | 36 (68%) | 6 (75%) | 3 |  |
|  | Yes | 82 (79%) | 2 (67%) | 1 |  | 17 (32%) | 2 (25%) | 1 |  |
| Invasion of lymphatic vessels, n (%) | |  |  |  | 1.000 |  |  |  | 0.710 |
|  | No | 32 (31%) | 1 (33%) | 0 |  | 26 (49%) | 3 (38%) | 0 |  |
|  | Yes | 72 (69%) | 2 (67%) | 1 |  | 27 (51%) | 5 (62%) | 4 |  |
| Invasion of blood vessels, n (%) | |  |  |  | 0.261 |  |  |  | 1.000 |
|  | No | 70 (67%) | 1 (33%) | 0 |  | 48 (91%) | 8 (100%) | 4 |  |
|  | Yes | 34 (33%) | 2 (67%) | 1 |  | 5 (9%) | 0 | 0 |  |
| Growth in peripancreatic fat, n (%) | |  |  |  | 1.000 |  |  |  | **0.042** |
|  | No | 23 (22%) | 0 | 0 |  | 31 (58%) | 8 (100%) | 4 |  |
|  | Yes | 81 (78%) | 3 (100%) | 1 |  | 22 (42%) | 0 | 0 |  |
| SATB1, n (%) | |  |  |  | 0.092 |  |  |  | 0.422 |
|  | Negative | 84 (81%) | 1 (33%) | 0 |  | 40 (75%) | 5 (62%) | 2 |  |
|  | Positive | 18 (17%) | 2 (67%) | 1 |  | 13 (25%) | 3 (38%) | 0 |  |
|  | Missing | 2 (2%) | 0 | 0 |  | 0 | 0 | 2 |  |
| Adjuvant chemotherapy, n (%) | |  |  |  | 1.000 |  |  |  | 0.319 |
|  | No adjuvant | 48 (46%) | 2 (67%) | 1 |  | 39 (74%) | 5 (62%) | 3 |  |
|  | 5FU-analogue | 8 (8%) | 0 | 0 |  | 5 (9%) | 0 | 0 |  |
|  | Gemcitabine | 43 (41%) | 1 (33%) | 0 |  | 5 (9%) | 1 (13%) | 1 |  |
|  | Gemcitabine + capecitabine | 2 (2%) | 0 | 0 |  | 1 (2%) | 0 | 0 |  |
|  | Oxaliplatin + 5-FU analogue | 1 (1%) | 0 | 0 |  | 2 (4%) | 2 (25%) | 0 |  |
|  | Gemcitabine + oxaliplatin | 2 (2%) | 0 | 0 |  | 1 (2%) | 0 | 0 |  |
| Recurrence | |  |  |  | 0.422 |  |  |  | 0.472 |
|  | No | 20 (19%) | 0 | 0 |  | 28 (53%) | 5 (62%) | 2 |  |
|  | Yes, local only | 29 (28%) | 0 | 0 |  | 3 (5%) | 1 (13%) | 0 |  |
|  | Yes, non-local | 55 (53%) | 3 (100%) | 1 |  | 22 (42%) | 2 (25%) | 2 |  |
| Included in survival analyses | |  |  |  | 1.000 |  |  |  | 1.000 |
|  | Yes | 103 (99%) | 3 (100%) | 1 |  | 51 (96%) | 8 (100%) | 4 |  |
|  | No | 1 (1%) | 0 | 0 |  | 2 (4%) | 0 | 0 |  |

M, median. IQR, interquartile range. Bold text indicates significant p-values.
